# Association between *MMP2-1306 C/T* polymorphism and prostate cancer susceptibility: a meta-analysis based on 3906 subjects

**DOI:** 10.18632/oncotarget.16972

**Published:** 2017-04-08

**Authors:** Kaiping Zhang, Xianguo Chen, Jun Zhou, Cheng Yang, Meng Zhang, Min Chao, Li Zhang, Chaozhao Liang

**Affiliations:** ^1^ Department of Urology, Anhui Provincial Children’s Hospital, Hefei, Anhui, China; ^2^ Department of Urology, The First Affiliated Hospital of Anhui Medical University and Institute of Urology, AHMU, Hefei, Anhui, China

**Keywords:** MMP2, polymorphism, prostate cancer, susceptibility, Gleason grading

## Abstract

Numerous investigations have addressed the correlation between *MMP2-1306C/T* polymorphism and prostate cancer (PCa) susceptibility. However, these conclusions were controversial. Thus, we conducted this current meta-analysis based on six studies from PubMed, Embase, Cochrane Library, China Biology Medicine disc (CBM), China National Knowledge Infrastructure (CNKI) up to October 21^st^, 2016. Odds ratios (ORs) with 95% confidence intervals (CIs) were calculated to evaluate the strength of the correlations. Additionally, different subgroup analyses and publication bias tests were performed. Eventually, six previous investigations consisted of 1920 cases and 1986 controls were identified and involved in this meta-analysis. Consequently, our evidence indicates a certain association between *MMP2-1306C/T* polymorphism and PCa risk among overall population (T vs C: OR = 1.12, 95% CI = 1.00-1.24, *P =* 0.040; TT+CT vs CC: OR = 1.16, 95% CI = 1.02-1.32, *P =* 0.026; respectively), as well as the subgroups of Asian population (T vs C: OR=1.48, 95% CI=1.13-1.94, *P=*0.004; TT+CT vs CC: OR = 1.66, 95% CI = 1.21-2.28, *P =* 0.002; respectively) and PCR-RFLP genotyped method (T vs C: OR = 1.58, 95% CI = 1.19-2.10, *P =* 0.001; TT+CT vs CC: OR = 1.71, 95% CI = 1.23-2.38, *P =* 0.001; respectively). However, no association was detected in *MMP2-1306C/T* polymorphism with Gleason grading or pathological stage of PCa. Our study indicates *MMP2-1306 C/T* polymorphism might increase PCa risk, particularly for Asian population. However, future studies comprising large cohort size from multicenter are required to confirm our conclusions.

## INTRODUCTION

Prostate cancer (PCa) is the primary cause of cancer-related death which seriously threatens psychological and physical health in older men. PCa characterized by high incidence and mortality rate has drawn extensive attention in clinic. An estimation of 2015 cancer statistics revealed 220,800 new patients and 27,540 new deaths assigned to PCa [1]. Whereas, the mechanisms of carcinogenesis were still largely unexplored. It is well-established that both environmental factors and genetic diversities have non-negligible influences upon prostatic carcinogenesis. Genome-wide association studies have detected different single nucleotide polymorphisms (SNPs) correlated with PCa susceptibility [2]. It has also been proven that additional independent SNPs at GWAS-identified loci were closely linked with PCa [3–5]. To our best knowledge, several genetic mutations including *nuclear factor kappa B (NF-κB), p53* along with *MMPs* have been identified, which raised major concerns regarding the roles of gene polymorphisms in carcinogenesis during past decades [6–8].

Matrix metalloproteinases (MMPs) belonged to extracellular matrix (ECM)-degrading enzymes participated in the mechanisms of inflammation and angiogenesis [9]. To date, preclinical researches revealed that these MMPs had great potential in cardiovascular disease therapy and diabetes biomarkers screening [10–11]. As for cancers, MMPs have been shown as characteristic sign of tumor invasion and prognosis [12]. Matrix metalloproteinase 2 (MMP2) has been extensively studied in MMPs. It is well known that MMP2 has a positive influence upon the cancerous progression which may be involved in tumor growth, invasion and metastasis [13]. The *MMP2* gene, located at Chromosome 16, is composed of 13 exons. Its polymorphism may be linked with different cancer risks owing to reduced enzymic activity [14]. Although several studies have shown that *MMP2-1306C/T* polymorphism might be closely associated with PCa development, the conclusions were not consistent yet, which results could be explained by the relatively small samples in each published study. Additionally, *MMP2* is upregulated in PCa, and higher abundance may indicate poorer prognosis [15]. Meta-analysis can explore the authentic and comprehensive results through incorporating all available evidences to get a relatively precise and accurate estimation using statistical software [16]. Herein, we conduct a meta-analysis to assess the possible correlations between *MMP2-1306C/T* polymorphism and PCa risk, which efforts should hold great promise in the clinical diagnosis and therapy for PCa.

## RESULTS

### Characteristics of eligible studies

**Figure 1:**
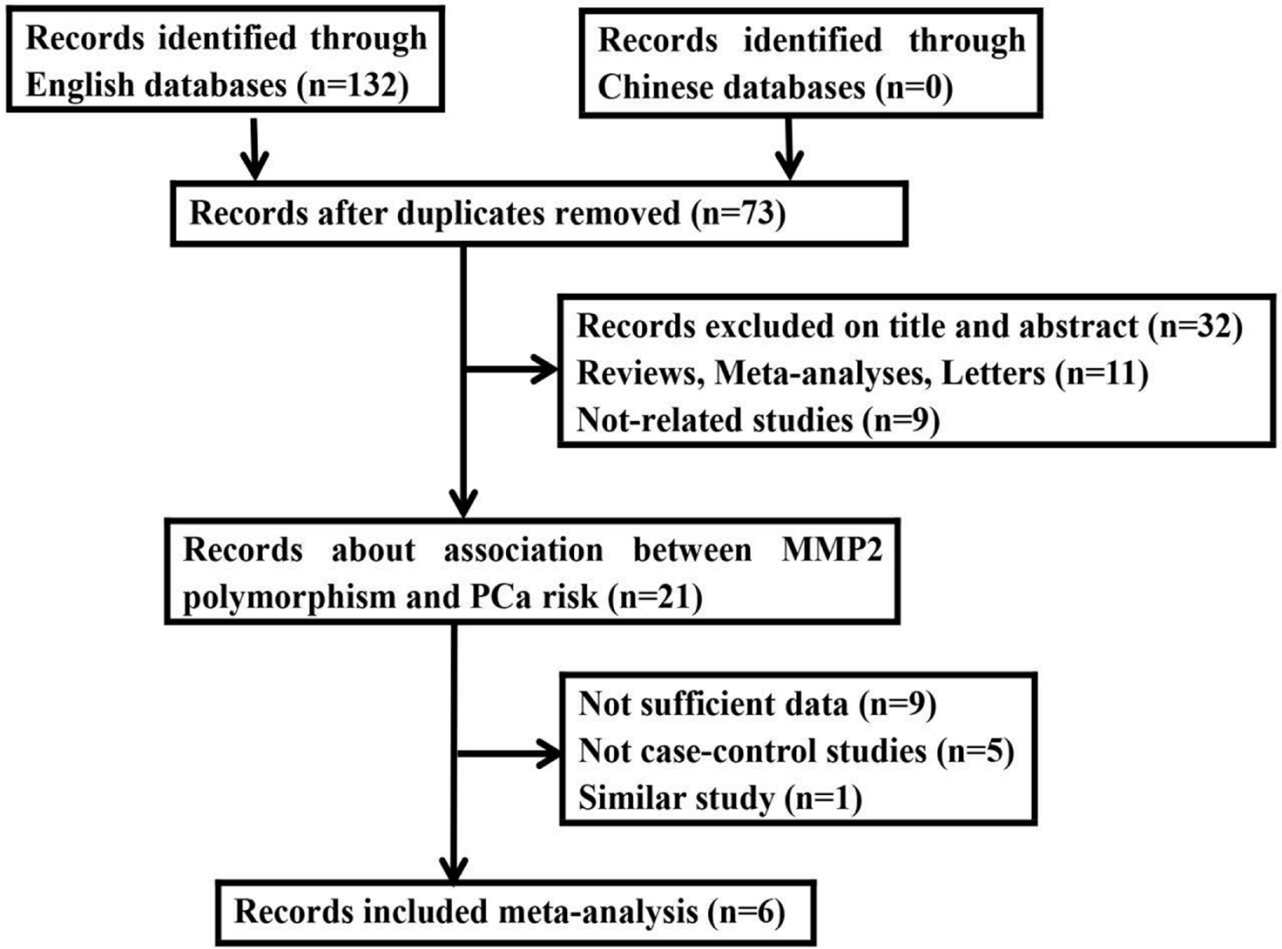
Flow diagram of the study selection process in the meta-analysis

Eventually, six studies consisted of 1920 cases and 1986 controls satisfied the eligible studies (Figure 1) [17–22]. Of the six studies, three White and three Asian population were estimated. The sample sizes ranged from 104 to 2867. Meanwhile, two Taqman, three PCR-RFLP and one HRM in genotyped approaches were introduced. Based on the control source, one was BPH and five were healthy PB as controls. The HWE of control were then evaluated among eligible studies. All PCa samples were histologically diagnosed. The relevant characteristics were shown in Table 1.

**Table 1:** Main characteristics of studies regarding the association between *MMP2-1306C/T* polymorphism and prostate cancer risk

Polymorphism	Authors	Year	Country	Ethnicity	Genotyped method	Source of control	Association	Case/Control	HWE`	NOS
*MMP2-1306C/T*	Shajarehpoor S et al. [17]	2016	Iran	Asian	HRM	PB	No	50/54	NO	7
	Adabi et al. [18]	2015	Iran	Asian	PCR-RFLP	BPH	No	101/137	Yes	7
	Yayksali et al. [19]	2014	Turkey	White	PCR-RFLP	PB	No	61/46	Yes	8
	Srivastava et al. [20]	2012	North India	Asian	PCR-RFLP	PB	Yes	190/200	Yes	8
	Dos Reis et al. [21]	2008	Brazil	White	TaqMan	PB	Yes	100/100	No	7
	Jacobs et al. [22]	2008	America	White	TaqMan	PB	Yes	1418/1449	Yes	7

PB population-based; BPH benign prostatic hyperplasia; HWE Hardy-Weinberg equilibrium of control; PCR-RFLP polymerase chain reaction and restriction fragment length polymorphism; HRM high-resolution melting analysis; NOS Newcastle-Ottawa Scale.

### Meta-analysis result

### Meta-analysis for *MMP2-1306C/T* polymorphism with PCa

**Figure 2:**
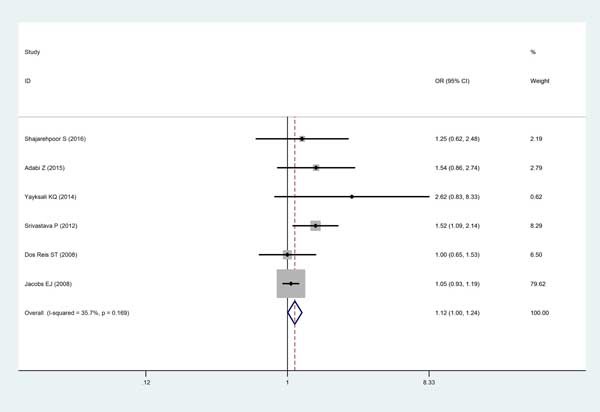
Forest plot for the meta-analysis of the association between ***MMP2-1306C/T*** polymorphism with PCa risk under allele comparison model with fixed-effects model. The squares and horizontal lines correspond to the study-specific OR and 95% CI. The area of the squares reflects the weight. The diamond represents the summary OR and 95% CI. CI = confidence interval, OR = odds ratio.

Finally, six studies consisted of 1920 cases and 1986 controls enrolled in this analysis using random- or fixed-effects model. Consequently, the pooled data indicated a certain association between *MMP2-1306C/T* polymorphism with PCa risk among overall population (T vs C: OR=1.12, 95% CI=1.00-1.24, *P=*0.040; TT+CT vs CC: OR=1.16, 95% CI =1.02-1.32, *P=*0.026; respectively) (Figure 2).

Subgroup analyses underlying the ethnicity, source of control and genotyped method were carried out. As a result, a certain association was detected in Asian population (T vs C: OR = 1.48, 95% CI = 1.13-1.94, *P =* 0.004; TT+CT vs CC: OR = 1.66, 95% CI = 1.21-2.28, *P =* 0.002; respectively) and in PCR-RFLP genotyped method (T vs C: OR=1.58, 95% CI= 1.19-2.10, *P* = 0.001; TT+CT vs CC: OR=1.71, 95% CI=1.23-2.38, *P* = 0.001; respectively). Conversely, there were no significant associations among other subgroups (Table 2).

**Table 2:** Stratified analysis of the *MMP2 -1306C/T* polymorphism and prostate cancer

Variables	Group	Case/ Control	Allele T vs C OR(95%CI)	*I^2^*	*P^h^*	Homozygous TT vs CC OR(95%CI)	*I^2^*	*P^h^*	Heterozygous TT vs CT OR(95%CI)	*I^2^*	*P^h^*	Recessive TT+CT vs CC OR(95%CI)	*I^2^*	*P^h^*	Dominant TT vsCC+CT OR(95%CI)	*I^2^*	*P^h^*
	Overall(6)	1920/1986	**1.12(1.00-1.24)**	35.7%	0.169	1.09(0.83-1.42)	0.0%	0.499	0.91(0.69-1.20)	43.2%	0.117	**1.16(1.02-1.32)**	39.5%	0.142	1.01(0.78-1.32)	12.2%	0.337
Ethnicity	Asian(3)	341/391	**1.48(1.13-1.94)**	0.0%	0.868	1.46(0.70-3.06)	0.0%	0.550	0.88(0.40-1.95)	0.0%	0.507	**1.66(1.21-2.28)**	0.0%	0.962	1.25(0.60-2.59)	0.0%	0.590
	White(3)	1579/1595	1.06(0.95-1.19)	18.3%	0.294	1.04(0.78-1.38)	17.7%	0.297	0.73(0.24-2.19)	73.1%	0.024	1.08(0.94-1.24)	11.5%	0.323	0.88(0.44-1.77)	53.1%	0.119
Sourcr of control	PB(5)	1819/1849	1.10(0.99-1.23)	38.9%	0.162	1.09(0.84-1.43)	3.6%	0.386	0.78(0.41-1.47)	51.8%	0.081	1.14(1.00-1.30)	39.8%	0.156	1.02(0.79-1.33)	26.6%	0.244
	BPH(1)	101/137	1.54(0.86-2.74)	/	/	0.51(0.02-12.6)	/	/	0.28(0.01-7.33)	/	/	1.72(0.92-3.20)	/	/	0.45(0.02-11.1)	/	/
Genotyped method	PCRRFLP(3)	352/383	**1.58(1.19-2.10)**	0.0%	0.672	2.06(0.87-4.88)	0.0%	0.553	1.24(0.51-2.99)	0.0%	0.509	**1.71(1.23-2.38)**	0.0%	0.952	1.75(0.75-4.11)	0.0%	0.529
	TaqMan(2)	1518/1549	1.05(0.94-1.18)	0.0%	0.817	1.01(0.76-1.35)	12.2%	0.286	0.60(0.18-2.00)	84.7%	0.010	1.07(0.93-1.23)	15.0%	0.278	0.81(0.40-1.61)	66.1%	0.086
	HRM(1)	50/54	1.25(0.62-2.48)	/	/	1.00(0.28-3.58)	/	/	0.53(0.12-2.42)	/	/	1.48(0.63-3.51)	/	/	0.89(0.25-3.12)	/	/
HWE	Yes(4)	1770/1832	1.34(0.98-1.82)	59.5%	0.060	1.16(0.87-1.56)	0.0%	0.416	1.06(0.78-1.43)	0.0%	0.069	1.39(0.97-1.98)	58.8%	0.063	1.13(0.85-1.50)	0.0%	0.523
NOS	≥7 (6)	1920/1986	**1.12(1.00-1.24)**	35.7%	0.169	1.09(0.83-1.42)	0.0%	0.499	0.91(0.69-1.20)	43.2%	0.117	**1.16(1.02-1.32)**	39.5%	0.142	1.01(0.78-1.32)	12.2%	0.337

OR odds ratio; CI confidence interval; P^h^ *P*-value of heterogeneity test; HWE Hardy-Weinberg equilibrium of control.

### Meta-analysis for *MMP2-1306C/T* polymorphism with Gleason grade and pathological stage

Among the previous eligible studies, only four studies have been performed to explore the correlations between *MMP2-1306C/T* polymorphism with different Gleason group grades [17–18, 20–21]. Patients were categorized into Gleason≥7 and Gleason<7. Overall, no statistically significant association was observed in *MMP2-1306C/T* polymorphism with any of the Gleason grading of PCa (T vs C: OR = 1.23, 95% CI = 0.85-1.78, *P =* 0.264; TT vs CC: OR = 1.88, 95% CI = 0.74-4.80, *P =* 0.185; TT vs CT: OR = 1.57, 95% CI = 0.60-4.11, *P =* 0.355; CT+TT vs CC: OR = 1.19, 95% CI = 0.75-1.87, *P =* 0.461; TT vs CC+CT: OR = 1.77, 95% CI = 0.72-4.35, *P =* 0.216; respectively). When analyzing pathological stage, there were only two related studies [17, 21]. Patients were divided into pT_3_ and pT_2_, and no association between *MMP2-1306C/T* polymorphism or pathological stage was addressed (T vs C: OR = 1.33, 95% CI = 0.43-4.10, *P =* 0.622; TT vs CC: OR = 1.39, 95% CI = 0.14-14.04, *P =* 0.782; TT vs CT: OR = 0.94, 95% CI = 0.30-2.93, *P =* 0.910; CT+TT vs CC: OR = 1.69, 95% CI = 0.51-5.65, *P =* 0.393; TT vs CC+CT: OR = 1.12, 95% CI = 0.18-6.93, *P =* 0.900; respectively). (Table 3)

**Table 3:** Meta-analysis for *MMP2-1306C/T* polymorphism with Gleason grade and pathological stage

Comparison	Gleason grade			pathological stage		
	OR(95%CI)	I^2^	P^h^	OR(95%CI)	I^2^	P^h^
T *vs* C	1.23(0.85-1.78)	1.8%	0.383	1.33(0.43-4.10)	71.7%	0.060
TT *vs* CC	1.88(0.74-4.80)	26.1%	0.258	1.39(0.14-14.04)	67.4%	0.782
TT *vs* CT	1.57(0.60-4.11)	23.6%	0.270	0.94(0.30-2.93)	10.8%	0.290
CT+TT *vs* CC	1.19(0.75-1.87)	0.0%	0.692	1.69(0.51-5.65)	59.2%	0.117
TT *vs* CC+CT	1.77(0.72-4.35)	29.7%	0.241	1.12(0.18-6.93)	52.6%	0.146

P^h^ P-value of heterogeneity test.

### Sensitivity analysis

**Figure 3:**
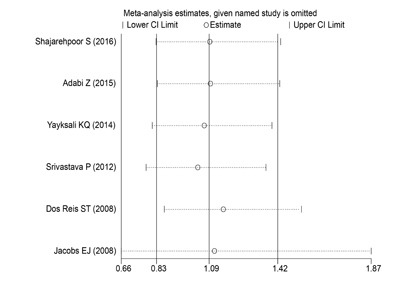
One-way sensitivity analysis of the ***MMP2-1306C/T*** polymorphism with PCa risk. Individually removed the studies and suggested that the results of this meta-analysis were stable.

Herein, each single study was deleted at a time to assess the specific effect of the individual data on the pooled ORs, and one-way sensitivity analysis revealed that pooled results were relatively stable (Figure 3).

### Publication bias evaluation

**Figure 4:**
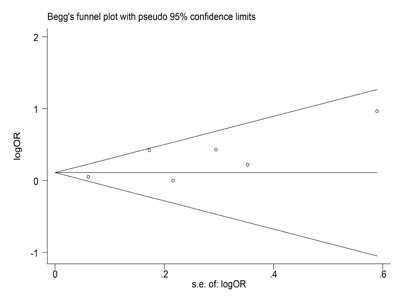
Begg's funnel plot to examine publication bias in allele of ***MMP2-1306C/T*** polymorphism. Each circle corresponds to one study, and indicated that no publication bias existed.

Begg's funnel plot indicated that publication bias was not found in allele of *MMP2-1306C/T* polymorphism (*P =* 0.452, Figure 4). Meanwhile, no publication bias was found in each subgroup of mata-analysis.

## DISCUSSION

Until now, investigations focused on the correlations between *MMP2-1306C/T* polymorphism with PCa were relatively rare and inconclusive. Meanwhile, small sample-sized studies lacking statistical power often result in apparently contradictory conclusions. Meta-analysis is an useful tool for providing convincing evidence as it could present inconsistent results from different investigations to obtain a relatively precise estimation. As far as we know, the current meta-analysis is the first try to comprehensively assess the correlation between *MMP2-1306C/T* gene polymorphism with PCa risk. Besides, the potential associations were explored in different subgroups. Consequently, the current meta-analysis revealed a certain relationship between *MMP2-1306C/T* polymorphism and PCa in overall group together with the subgroup of Asian population and PCR-RFLP genotyped method. However, no significant association was observed in *MMP2-1306C/T* polymorphism with Gleason grading or pathological stage of PCa.

Due to significant heterogeneity of the current meta-analysis, careful interpretation and search for influencing factors were required. Through different subgroups analyses, differences in the ethnicity and genotyped method should be considered as potential sources of heterogeneity. Additionally, sample size may have an impact on heterogeneity. It is well-established that formalin-fixation and/or prolonged storage could elicit damage to nucleic acids, further conferring considerable limitation on results [23–24]. Besides, other undiscovered factors should also be taken into consideration in advanced researches.

Recently, the associations between *MMP2* polymorphisms with cancers have been extensively explored in published meta-analyses. Whereas, these results differed greatly in various types of cancer. For instance, no associations were identified between *MMP-2-1306C/T* polymorphism with susceptibility of breast cancer, colorectal cancer and gastric cancer, respectively in previous meta-analyses [25–27]. In addition, *MMP1, MMP3* and *MMP9* polymorphisms were not linked with colorectal cancer susceptibility [27]. Moreover, no significant relationships of *MMP-2-1306* and *-735 C/T* polymorphisms were found with coronary artery disease risk [28] Whereas, other meta-analysis illustrated that the *MMP-2-1306* polymorphism was significantly related to bladder cancer and head and neck cancer (HNC) risk among overall population [29–30], especially for Asian populations who were diagnosed as lung cancer [31]. The aforementioned contradictory results could be clarified that for certain population, distinct genes, loci within identical genes and various polymorphisms at same locus may affect different cancer susceptibilities [32]. Therefore, we performed the current meta-analysis to explore the potential correlation between *MMP2-1306 C/T* polymorphism with PCa susceptibility in overall population and corresponding subgroups

MMPs are endopeptidases capable to degrade collagens from extracellular matrix (ECM). They are essential for proliferation, differentiation, morphogenesis, tissue remodeling and repair [33]. MMPs are engaged in cell cycle checkpoints control, cell adhesion and genomic instability, whose activity could be depressed by tissue inhibitors of metalloproteinases (TIMPs) [34]. MMPs and TIMPs could modulate the remodeling of ECM. The imbalance between MMPs and TIMPs may result in pathological processes such as arthritis and cancer [35]. Meanwhile, MMPs could alter the cellular microenvironment, which could facilitate tumor initiation and development [36]. Thus, excessive expression of MMPs may play crucial roles in cancer by facilitating ECM degradation. Notably, MMPs and TIMPs were proven to contribute to PCa risk. Circulating levels affected by single nucleotide polymorphisms (SNPs) of *MMPs* gene promoter might lead to relevant biological responses. It has reported that *MMP-9*, rather than *MMP-1* polymorphism variants were associated with pathological parameters in predicting the clinical outcome of prostate cancer patients [37–39]. As for MMP-2, it may be a poor prognosis indicator of PCa on account of serum/tissue over-expression in higher Gleason scores and cancerous invasion [40]. Previous study found that patients with CT genotype as well as T allele were significantly associated with 1.68-fold and 1.52-fold increased risk of PCa [41]. However, it was also reported that the genotype CT of *MMP2* was related to lower levels of *MMP2* mRNA and surprisingly lower circulating levels of *MMP2* were related to more aggressive PCa in culture cell lines. Thus, prostate cancer may be conversely responsible for lower gene/protein expression of *MMP2* [42]. In view of multiple lines of contradictory results, we tried to explore the potential relationship in the current meta-analysis. To sum up, as most studies have presented definite relationships of *MMP* polymorphisms in various types of cancer [43–45], MMPs may become putative therapeutic targets for cancer [46].

Actually, our meta-analysis has its limitations. Firstly, it is subjected to recall or selection bias of retrospective study. Secondly, only published studies might not provide sufficient evidences in this meta-analysis. Finally, our conclusion was checked by crude estimation rather than adjusted data. Therefore, other risk factors such as environmental effects, genetic factors and environment-gene interactions should also be taken into consideration in advanced researches. Meanwhile, the heterogeneity indicated there were potential or undiscovered factors in included publications. Moreover, several controls from eligible investigations didn't conform to HWE, which may also influence the ultimate conclusion. Anyway, in spite of aforementioned limitations, a certain relationship of *MMP2-1306 C/T* polymorphism in PCa risk was identified in current meta-analysis.

In conclusion, the current study is the first original meta-analysis to address the correlation between the *MMP2-1306 C/T* polymorphism and PCa susceptibility. A marginally significant association was explored in overall population as well as the subgroups of Asian population and PCR-RFLP genotyped method. It presented that *MMP2-1306 C/T* polymorphism might increase PCa risk to some extent. No association was detected in *MMP2-1306 C/T* polymorphism with any of the Gleason grading or pathological stage of PCa. However, in the future, well-designed prospective studies with large cohort size and various SNPs are urgently necessary to verify our current findings.

## MATERIALS AND METHODS

### Ethics statement

Meta-Analysis of Observational Studies in Epidemiology (MOOSE) directions for reporting were used to perform the current meta-analysis [47]. No patient's privacy or clinical samples were involved in this study, hence the ethical approval was not required.

### Identification and eligibility of relevant studies

Literature resources including PubMed, Cochrane Library, Embase, CBM and CNK were searched for eligible literatures, using the terms (“matrix metalloproteinases” or “matrix metalloproteinases 2” or “MMPs” or “MMP” or “MMP2”), (“prostatic cancer” or “prostate cancer” or “prostatic carcinoma” or “prostate carcinoma” or “PCa”) and (“polymorphism” or “variant” or “mutation”). Last search of current investigation was updated on October 21^st^, 2016. There were no language restrictions. We identified other relevant articles by scanning all retrieved articles and reviews. Meanwhile, we treated them independently if different ethnicities were found in reported articles.

### Inclusion and exclusion criteria

Studies followed the three criteria could be identified: (1) all included studies belonged to case-control or cohort studies; (2) relevant data to evaluate the correlations between *MMP2* polymorphisms with PCa risk were available; (3) PCa was histologically confirmed. Studies met the following three criteria were excluded: (1) the available data regarding about associations was absent; (2) similar or duplicate study (When the same or similar cohort was applied, after careful examination, the most complete information was included); (3) other types of articles including reviews or abstracts.

### Data extraction

In the light of inclusion and exclusion criteria, we extracted the relevant information from each eligible publication. If disagreements were noticed, we were clearly open to discuss by each other (K.Z. and M.C.), or reviewed by a third author (X.C.).

The data on first author, race, study country, number of case and control, publication year, genotyped method, study design, control source, whether there were certain associations between the paired groups, and *P* value from Hardy-Weinberg equilibrium of control were collected by two authors independently. The Newcastle-Ottawa Scale consisted of selection, comparability of the groups and ascertainment of exposure was introduced to evaluate the included publication's quality. The NOS scores were 0 to 10 stars. If one included study obtained no less than 7 stars, it could be regarded as high-quality [48]. We have not contacted any author of the original researches even though the essential information could not be available. Besides, ethnicities were stratified into two groups: White and Asian population. Source of controls mainly derived from healthy population-based (PB) and benign prostatic hyperplasia (BPH) population. Genetyped methods were divided into PCR-RFLP, Taqman and HRM.

### Statistical analysis

We explored the relationship *MMP2-1306C/T* polymorphism and PCa risk by applying STATA software (Version 12.0, Stata Corporation, TX). OR and 95% CI were calculated for assessing the concrete relationships between *MMP2-1306C/T* polymorphisms and PCa susceptibility. Varying models for genotyping, including allele comparison, dominant, recessive, homozygote and heterozygote models were applied to determine the associations with PCa risk. Meanwhile, the heterogeneity has been assessed via chi-square-based Q and I^2^ test across studies (no heterogeneity I^2^<25%, moderate heterogeneity I^2^=25%-50%, extreme heterogeneity I^2^>50%) [49]. In case of extreme heterogeneity (I^2^>50% or *P <* 0.01 for Q test), we used random-effects (DerSimonian and Laird method) model [50]. Otherwise, fixed-effects (Mantel-Haenszel method) model was introduced [51].

One-way sensitivity analyses individually removed publications in meta-analysis were conducted to assess results’ stability. It mainly explored the impact of specific study upon mixed OR.

The Begg's funnel plots where logOR was plotted against SE. *P* value less than 0.05 indicated that there was a bias of study [52]. Additionally, different subgroups consisted of ethnicity, control source and genotyped approach were conducted.
